# Parameter-less approaches for interpreting dynamic cellular response

**DOI:** 10.1186/1754-1611-8-23

**Published:** 2014-08-19

**Authors:** Kumar Selvarajoo

**Affiliations:** 1 Institute for Advanced Biosciences, Keio University, Tsuruoka, Yamagata, Japan; 2 Systems Biology Program, Graduate School of Media and Governance, Keio University, Fujisawa, Japan

**Keywords:** Biological networks, Non-parametric, Cell signaling, Immune response, Gene expression

## Abstract

Cellular response such as cell signaling is an integral part of information processing in biology. Upon receptor stimulation, numerous intracellular molecules are invoked to trigger the transcription of genes for specific biological purposes, such as growth, differentiation, apoptosis or immune response. How complex are such specialized and sophisticated machinery? Computational modeling is an important tool for investigating dynamic cellular behaviors. Here, I focus on certain types of key signaling pathways that can be interpreted well using simple physical rules based on Boolean logic and linear superposition of response terms. From the examples shown, it is conceivable that for small-scale network modeling, reaction topology, rather than parameter values, is crucial for understanding population-wide cellular behaviors. For large-scale response, non-parametric statistical approaches have proven valuable for revealing emergent properties.

## 

The interpretation of dynamic cellular processes is indispensable for biological research. Especially in the last two decades, there have been tremendous efforts that were aimed at understanding complex biological networks in different cell types to various kinds of stimulations or perturbations, and in disease conditions using systems biology approaches. What sort of models do we need to conceptualize biological networks for interpreting or predicting dynamic responses?

In the early 1900s Victor Henri, Leonor Michaelis and Maud Menten thoroughly investigated enzymatic reactions *in vitro*, and developed the hyperbolic rate equation that we now popularly call the Michaelis-Menten enzyme kinetics. This is a more sophisticated form of mass-action type reaction, considering the saturation of kinetics at higher substrate concentrations instead of ever increasing profile for the latter. Subsequent work on this basic principle led to the extension of the kinetics to represent more complex scenarios, such as multi-substrate ping-pong and ternary-complex mechanisms [1].

As the development of computing power progressed significantly in the 1960s, there have been numerous efforts to model complete biological pathway modules, such as the glycolysis, using enzyme kinetic equations with the aim of estimating parameter values by fitting to steady-state concentration levels of metabolites. However, the more truthful abstraction of enzymatic complexity resulted in a dilemma where increased accuracy required increased knowledge of many parameters that were too difficult to obtain precisely. If parameter values are not accurately determined, the enzymatic reaction models will not be able to recapitulate experimental outcome reasonably well.

Most, if not all, studies adopting *in vitro* experiments determine the parameter values of reaction species for computational modeling from an artificial environment where the species are deliberately purified from its physiologic neighbors. This is because, until today, the *in vivo* kinetic parameters cannot be reliably measured using the current experimental technologies. Notably, there have been various reports that claim the kinetic parameters determined through *in vitro* and *in vivo* experiments can differ by several orders of magnitudes [2]. As a result, when combining these errors into the model, the final predictions could differ by several orders of magnitude. For example, the steady-state concentration of the glycolytic metabolite 3-phosphoglycerate in *Trypanosoma brucei* was under-predicted by an order of 7 [3].

The difficulty of accurately determining parameter values led to the development of non-parametric approaches such as the flux-balance analysis (FBA) [4]. Here, only the reaction topologies or stoichiometry of the network and steady-state levels are required to be known. Constraints are introduced by the stoichiometric coefficients in the system for the optimization of certain biological function, such as growth or production of certain compounds. Although, the FBA requires the assumption that metabolite concentrations remain at steady-states for analysis, it has been successfully used to interpret important physiological functions of a living cell. For example, Palsson and colleagues experimentally verified their prediction for the primary carbon source and oxygen uptake rates for maximal cellular growth in *E. coli*[5]. So, why is such simple steady-state method relying on stoichiometry of reactions make useful predictions? (Note that FBA requires the network topology to be largely known, as is the case for metabolic networks. For signaling pathways, where the detailed role of numerous molecules are still incomplete, FBA has limited application).

In a pioneering work on understanding the complex dynamics of bacterial chemotaxis, Leibler and colleagues created a highly simplified two-state mass-action model of *E. coli* chemotactic network [6]. Using the model, and subsequently with experiments [7], they showed that the adaptation precision of bacterial chemotaxis was insensitive to the large variation of its network parameter values. This mechanism, therefore, allows *E. coli* to display robust behavior to a wide range of attractant and repellent concentrations. However, at the same time, other properties, such as adaptation time and steady-state tumbling frequency, were variable to the stimulant concentration. Overall, their work demonstrated that bacterial adaptation property is a consequence of network’s connectivity and does not require the precision of parameter values. This work is a milestone paper that indicates complex biological phenomena can be understood using simple models that are not sensitive to parameter values.

The observation of simplicity in what appears to be highly dynamic and complex can have profound benefits in understanding and controlling disease conditions. Our research has focused on cell signaling dynamics of innate immune response and cancer cell survival. Over the last decade, we adopted systems biology approaches to study toll-like receptor (TLR) signaling [8-10], tumor necrosis factor (TNF) signaling [11] and TNF-related apoptosis-inducing ligand (TRAIL) signaling [12], from receptor stimulation through downstream gene expressions, via transcription factor activations.

The strategy was to first create a dynamic computational model based on current known pathways of a signaling process. Next, first-order response (mass-action) equation was used to represent each signaling reaction or process (protein binding, complex formation, ubiquitination, etc.). Subsequently, the model parameters were chosen to fit wildtype experimental dynamics, and compared with mutant cells for reliability of the models and their parameters [13]. When a single model is unable to simulate multiple experimental conditions, the model’s topology was allowed to be modified, using *response rules*, in accordance with the law of signaling flux conservation [9-13]. This is simply because we do not yet possess complete knowledge of all signaling reactions or molecules involved.

Notably, for all the complex signaling processes that we have investigated so far, we were successful to predict novel signaling features, such as missing intermediates, crosstalk mechanisms, feedback loops [8,11,12], and identify novel targets for controlling proinflammatory response [11] and cancer apoptosis [12]. All the predictions have been experimentally validated [9,11,14,15]. So why do simple models utilizing first-order response equations sufficient to produce insightful results of a complex system?

Firstly, the main reason for us to utilize first-order terms is due to the experimental observation of deterministic response waves of signal transduction within the period of investigations, usually up to 1-2 h after stimulation. That is, stimulating cell population in a dish with respective ligands resulted, in general, to dynamic activation response of intracellular proteins that followed gradual increase from their initial state to reach peak activation levels and, subsequently, decay to their original state (Figure 1A). Such responses are observed for the first round of response waves of myriad signaling species (Figure 1B). Although the kinetics could vary slightly from sample to sample, the general average response profiles are very well reproducible. In other words, regardless of how complex a signaling topology might be, the species’ average dynamic responses followed deterministic formation and depletion waves [13,17,18].

**Figure 1:**
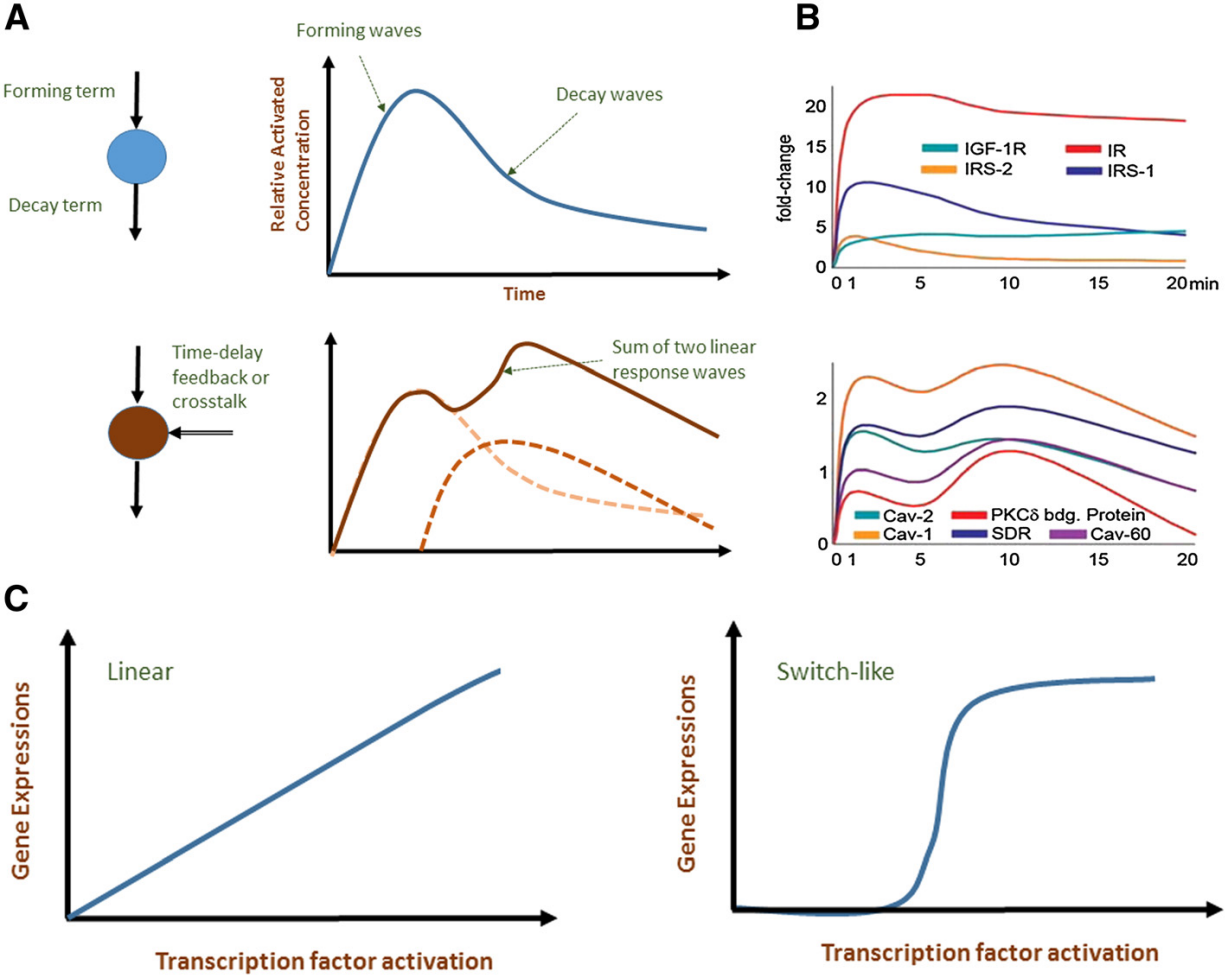
**The observation of linear response waves. A)** Schematic of activated signaling species, such as protein binding and gene expressions, with respect to time following formation and decay waves. Top panel represents a simple linear cascade with single wave. Bottom panel illustrates two linear waves superposed, as a consequence of an additional time-delay formation term. This may arise from feedback or crosstalk mechanisms. **B)** Quantitative dynamics of key molecules in insulin signaling pathway, showing similar dynamics to schematic in **A)**. Figures adapted from [16]. **C)** Schematic of linear and switch-like relationship between transcription factor concentration and gene expressions.

Secondly, it can be shown, theoretically, that no matter how complex or non-linear the signaling system is, the dynamic response can be approximated using first-order terms if the perturbation levels are small. Consider the general form of a complex kinetic equation: $\frac{\partial X}{\partial t}=F\left(X\right)$. *F* can be any non-linear function constituting of reaction and diffusion terms of species *X*. In engineering, for relative changes and when insufficient information is available, such systems are often carefully linearized using power or Talyor series ( $\frac{\partial \delta X}{\partial t}={\left(\frac{\partial F\left(X\right)}{\partial X}\right|}_{X_{a}}\delta X+{\left(\frac{\partial F^{2}\left(X\right)}{2!\partial X^{2}}\right|}_{X_{a}}\delta X^{2}+\ldots$, where *X = X*_*a*_ is the point for linearization). Given a small perturbation, the higher order terms become less significant, leaving only the first-order term as the dominant factor. Note that the linearization techniques are approximate methods to understand general behaviors and, in many cases, cannot be used to interpret detailed mechanisms of response.

In light of this linear response hypothesis, it is noteworthy to quote the recent findings of two relevant works, that studied the relationships between the transcription factors and gene expressions in TNF-induced [19] and Msn2 overexpressed [20] stress response. Collectively, they found that increasing transcription factor concentration resulted in graded gene expressions that approximately followed a linear relationship (Figure 1C). Although it is known that many transcription factors produce switch-like or digital relationship due to cooperativity in DNA binding, the stress response transcription factors have shown simple graded behavior. This finding may justify that certain key cellular processes, such as the immune response, may be guided by linear response through the signaling cascades. Taken together, it appears that linear response, as a governing principle, is key to invoke precise and optimal response when living cells are faced with immediate threats.

In other studies, even without the need to know graded response, binary (ON/OFF) state approaches have yielded fascinating results in understanding cell signaling. One notable study developed discrete Boolean network modeling to investigate the survival mechanism of cytotoxic T lymphocytes (CTL) in T cell large granular lymphoctye (T-LGL) leukemia [21]. Loughran and colleagues created a T-LGL survival signaling model with 58 nodes, representing molecular species, and 123 edges, representing causal interactions between the species. Using the model, they identified the most significant interactions for activating CTL in disease state compared to normal. Subsequent experiments confirmed their model predictions.

It is conceivable that the arrival to parameter-less approaches may be unrealistic in the realm of complex systems, where non-linear factors and stochastic effects can cause even small variation in perturbations to produce diverse multistable outcomes or oscillatory patterns. Such is the case observed for cell fate decisions where a single fertilized egg can diversify into distinct cell lineages or a bacteria being able to change fate under nutrient-deficient condition [22]. To model such complexity, dynamical systems theory adopting non-linear equations may possibly be used [23]. Also, for understanding self-organizing behaviors such as biological clocks/rhythms, spatial patterns, Hopf bifurcation or other non-linear dynamics, Goodwin, Brusselator, and Lotka–Volterra equations have been widely adopted [24-26]. However, these models require the precision of parameter values and most often reproduce only the general behavior of complex biological responses in one (wildtype) condition.

Another issue to consider is the scale of networks. So far, biological modules or network modeling that have been successfully used consist of molecular species that are relatively small, in the order of tens or a few hundreds. However, the living system invokes response of thousands of species and such large-scale studies probably require different approaches. One common strategy used to tackle large-scale effects is to use statistical techniques that investigate regression or correlation between species and samples, or apply clustering techniques to identify groups of genes with similar temporal or functional behaviors [27,28]. These methods have been instrumental in revealing emergent behaviors, for example, the observation of collective oscillations of numerous cell cycle independent specific metabolic cycle genes in *Saccharomyces cerevisiae*[29,30], and the collective genome-wide expression dynamics, including lowly expressed genes, for innate immune response [31,32] and neutrophil cell differentiation [33,34]. For classifying distinct cancer types for targeted therapy, self-organizing maps on high-dimensional gene expression data have been highly useful [35]. Thus, non-parametric statistical works on high throughput gene expression datasets have been crucial in showing emergent self-organized behaviors in cell populations.

In the future, non-parametric autonomous Boolean circuits, that have been recently shown to generate chaos, with multiple attractor states through time-delayed feedback loops in physical signal propagation [36,37], may also be investigated for biological systems. These could, especially, be valuable for the application of cell signaling related to non-linear cell fate decisions or disease formation.
